# Defining muscle-invasive bladder cancer immunotypes by introducing tumor mutation burden, CD8+ T cells, and molecular subtypes

**DOI:** 10.1186/s41065-020-00165-7

**Published:** 2021-01-02

**Authors:** Zihao Chen, Guojun Liu, Guoqing Liu, Mikhail A. Bolkov, Khyber Shinwari, Irina A. Tuzankina, Valery A. Chereshnev, Zhifeng Wang

**Affiliations:** 1 School of Chinese Medicine, Faculty of Medicine, The Chinese University of Hong Kong, Hong Kong SAR, China; 2 Department of Medical Biochemistry and Biophysics, Institute of Natural Sciences and Mathematics, Ural Federal University, Ekaterinburg, 620000 Russia; 3 School of Life Science and Technology, Inner Mongolia University of Science and Technology, Baotou, 014010 China; 4 Department of immunochemistry, Institute of Chemical Engineering, Ural Federal University, Ekaterinburg, 620000 Russia; 5 Institute of Immunology and Physiology, Ural Branch of the Russian Academy of Sciences, Ekaterinburg, 620000 Russia; 6 Department of Urology, Henan Provincial People’s Hospital, Zhengzhou, 450003 China

**Keywords:** TMB, CD8+ T cells, Molecular subtype, Immunotherapy, MIBC, Immunotype

## Abstract

Immunotherapy, especially anti-PD-1, is becoming a pillar of modern muscle-invasive bladder cancer (MIBC) treatment. However, the objective response rates (ORR) are relatively low due to the lack of precise biomarkers to select patients. Herein, the molecular subtype, tumor mutation burden (TMB), and CD8+ T cells were calculated by the gene expression and mutation profiles of MIBC patients. MIBC immunotypes were constructed using clustering analysis based on tumor mutation burden, CD8+ T cells, and molecular subtypes. Mutated genes, enriched functional KEGG pathways and GO terms, and co-expressed network-specific hub genes have been identified. We demonstrated that ORR of immunotype A patients identified by molecular subtype, CD8+ T cells, and TMB is about 36% predictable. *PIK3CA*, *RB1*, *FGFR3*, *KMT2C*, *MACF1*, *RYR2*, and *EP300* are differentially mutated among three immunotypes. Pathways such as ECM-receptor interaction, PI3K-Akt signaling pathway, and TGF-beta signaling pathway are top-ranked in enrichment analysis. Low expression of *ACTA2* was associated with the MIBC survival benefit. The current study constructs a model that could identify suitable MIBC patients for immunotherapy, and it is an important step forward to the personalized treatment of bladder cancers.

**Supplementary Information:**

The online version contains supplementary material available at 10.1186/s41065-020-00165-7.

## Introduction

As one of the most common genitourinary malignancies, bladder cancer (BLCA) affects about 549,000 people globally with 200,000 deaths, in 2018 [1]. A quarter of patients with BLCA are muscle-invasive bladder cancer (MIBC) with a higher risk of metastasis, in which cancer cells may spread to regional pelvic lymph nodes and/or visceral sites, causing the disease incurable [2]. Radical cystectomy (RC) with neoadjuvant cisplatin-based chemotherapy (NAC) is the standard first-line multimodal treatment for MIBC patients, yet roughly 60% of MIBC patients do not have a significant treatment response [3]. In addition, due to toxicity, many patients are unsuitable or unwilling to receive cisplatin treatment [4].

Recently, great progress has been made in the field of anti-cancer immunotherapy. The utilization of immune checkpoint inhibitors such as anti-PD-1/PD-L1 relatively improved the treatment of MIBC [5]. To be specific, several *PD-1*/*PD-L1* inhibitors, such as Pembrolizumab, Atezolizumab, Durvalumab, Nivolumab, and Avelumab, are approved by the US Food and Drug Administration (FDA) in clinical use for advanced MIBC patients, who failed prior platinum-based chemotherapy [6]. Although the efficacy of immunotherapy has been demonstrated, the number of MIBC patients who respond to immunotherapy is limited. In clinical trials, Nivolumab, a human IgG4 *PD-1* antibody, has only below 20% of durable response rate in patients with metastatic or locally advanced urothelial carcinoma [7].

In order to identify appropriate candidates for immunotherapy and tailor immunotherapy treatment strategies, some biomarkers are being developed based on tumor *PD-L1* expression, tumor mutational burden (TMB), tumor-infiltrating lymphocytes (TILs), and several other factors [8]. As shown by several studies, patients with high tumor *PD-L1* levels showed better response rates to immunotherapy and longer survival [9]. Immunotherapy acts in part by reinvigorating a pre-existing tumor immune response, and the density of TILs, especially CD8+ T cells, is a strong positive prognostic indicator [10]. TMB refers to the number of somatic mutations per 1 million bases [11], and tumor cells with high TMB may have more neoantigens which could be recognized by T cells and incite an anti-tumor response [12]. More recently, attempts to jointly use TMB and tumor-infiltrating T cells to identify *PD-1* antibody responders, that has been reported in 22 different tumor types [13]. Apart from these biomarkers, molecular subtype has been considered to be a novel approach for identifying candidates for immunotherapy in different studies [14–16]. In MIBC, patients can be mainly and obviously classified into luminal and basal subtypes by RNA expression profiling, where the patients in basal subtype are more associated with the epithelial-mesenchymal transition (EMT), immune-related pathways, and unfavorable survival than luminal subtype [17–19]. However, more investigations are needed to confirm the role of molecular subtypes in predicting the treatment response of MIBC patients to immunotherapy.

In the era of precision immunotherapy, it is of utmost importance to construct immunotype model that could indicate the response rate to immunotherapy and to identify mediators that play key determining roles. Models and biomarkers could influence immunotherapy response, personalize cancer treatment, minimize side effects, decrease treatment cost, and avoid immune-related adverse events.

To tackle the above-mentioned problems, the current study attempts to (i) construct superior immunotype in MIBC patients by TMB, MIBC-specific immune cell infiltration, and molecular subtype, and (ii) predict the biomarker that can characterize the immunotype. For those immunotypes, the corresponding mutational genes, enriched functional KEGG pathways and GO terms, and hub genes in the co-expression network were proposed.

## Materials and methods

### Data acquisition

A level-3 RNA-sequencing data plus clinical information were obtained from The Cancer Genome Atlas (TCGA) data portal, and the corresponding mutation annotation file (MAF) was retrieved using the ‘TCGAbiolinks’ R package by specifying the “mutect” pipeline [20]. IMvigor210 II trial, a cohort of 348 MIBC patients treated with Atezolizumab (*PD-L1* inhibitor), was collected from the previous study, including the gene expression data, clinical information, and immune therapy response records [21]. Immune cell proportions (such as B cells, dendritic cells, macrophages, neutrophils, NK cells, CD4+ T cells, and CD8+ T cells) against each sample were calculated by CIBERSORT algorithm, with 1000 permutations [22]. Only mutations in coding genes were retained and the TMB was calculated as follows:

$$ TMB=\frac{M}{38} $$

where *M* is the total number of mutations in each sample, and 38 represents the number of megabases of human exome.

### Clustering analysis

The molecular subtype data (like basal and luminal classification) of 403 patients used in this study were obtained from our previous study, and luminal and basal subtypes were transformed to 0 and 1, respectively [19]. In terms of CD8+ T cells and TMB, the values of CD8+ T cells and TMB of each patient were assigned to numbers 0 and 1 based on their median value (0: lower than median value; 1: higher than median value). Next, we constructed the immunotypes following the basic idea of Cluster of Cluster (CoC) [23] analysis based on the following 3 platforms: molecular subtype, TMB, and CD8+ T cells. Briefly, subgroups defined from each platform were coded into a series of indicator variables, resulting in a matrix of 1 and 0 (*M*_*i,j*_) whose each element *E*_*i,j*_ can be defined as

$$ {E}_{i,j}=\left\{{}_{0,\kern2.5em otherwise}^{1,\kern3em \mathrm{if}\kern0.5em {E}_{i,j\kern0.5em }\kern0.5em \in \kern0.5em {C}_j}\right. $$

where *C*_*j*_ represent the clusters from each platform (i.e. subtype_1, subtype_2, TMB_1, TMB_2, CD8 + _T_cell_1, CD8 + _T_cell_2, etc) and *i* is in the range from 1 to 403. Next, a well-known consensus clustering (CC) algorithm was performed on the *M*_*i,j*_ matrix using the “ConsensusClusterPlus” package. The optimal number of clusters (K) was estimated by commonly used methods including cumulative distribution function (CDF) and relative change in area under CDF curve [24].

### Training a random forest model and its validation

Random forest (RF) model, one of the most popular machine learning methods used for supervised learning, was used to train on the TCGA dataset and validate on the IMvigor210 dataset. Specifically, on the basis of the 736 immune-related gene expression profile in the TCGA dataset, we trained the regression RF model that predicts the immunotype A, B, and C, with the optimal parameters of “*mtry* = 3” and “*ntree* = 500”. After completing the training process, the RF model was used to predict immunotypes based on the expression profile of the immune-related genes on the IMvigor210 dataset. Seven hundred thirty-six immune-related gene list was downloaded from the previous study (https://www.ncbi.nlm.nih.gov/geo/query/acc.cgi?acc=GPL25507).

### Gene network analysis

To reduce outliers and simplify the calculation burden, we first retained 4677 prognostic-associated mRNAs selected by the univariate Cox proportional hazards regression (*P* < 0.05). A weighted correlation network analysis (WGCNA) was further performed on prognostic-associated mRNAs to construct scale-free gene co-expression networks, by using the R package “WGCNA”, with min-ModuleSize of 20, mergeCutHeight of 0.25, and unsigned topological overlap measure (TOM) [25]. An appropriate soft-threshold power was selected according to standard scale-free distribution (scale independence and mean connectivity). The module eigengenes (MEs) based on the first principal component were calculated for each module to measure the correlation between modules and clinical information including immunotype. The experimental protein-protein interactions (PPIs) data for genes within the immunotype-related module were retrieved from the STRING database, by specifying the interaction score of more than 0.4 (http://string-db.org/). The network was visualized within Cytoscape software (version 3.5.1.), and the hub genes were selected based on the Matthews correlation coefficient (MCC) score by using “cytoHubba” plug.

### Statistical analysis

The Kaplan–Meier model available in R package “survival” was used to calculate overall survival (OS) plus log-rank test to compare survival time between different groups. Kyoto Encyclopedia of Genes and Genomes (KEGG) and Gene Ontology (GO) enrichment analysis were performed by R package “clusterprofiler”, with the cut-off value of FDR less than 0.05 [26]. The MAF data was analyzed using an R package ‘maftools’ [27]. Pearson correlation coefficient (PCC) analysis was used to evaluate correlations between continuous variables (e.g. TMB, CD8+ T cells, genes expression). For analyzing the levels of CD8 + T cells and TMB in different groups, the Wilcoxon rank-sum test was used to compare the average between the two groups, and the Kruskal-Wallis test was used for the comparison of more than two groups. Clinical characteristics were estimated by univariate Cox regression followed by multivariate Cox regression to identify independent prognostic factors. The chi-square independence test evaluates if two categorical variables such as immunotypes and pathological stages are related in any way. All statistical analyses in this study was performed using Python version 3.7.1 (https://www.python.org/) or R version 3.4.3 (https://www.r-project.org/), unless mentioned otherwise. Conventionally, the *P* < 0.05 was regarded as statistically significant.

## Results

### Prognostic importance of CD8+ T cells and TMB

**Fig. 1:**
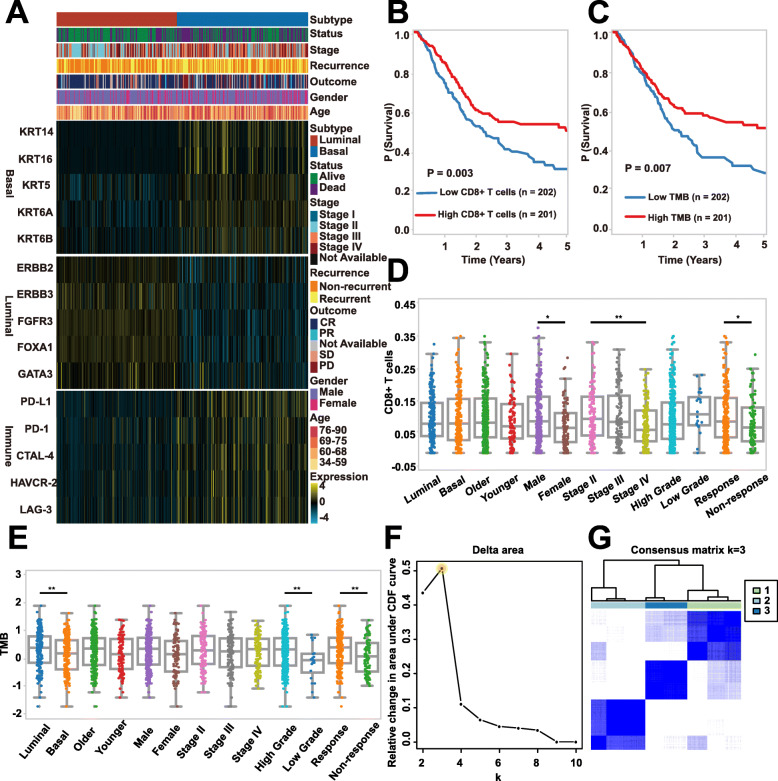
Classifying MIBC patients into three immunotypes. **a** The heat map shows the clinical features of the two molecular subtypes as well as the expression distribution of molecular subtype and immune-related biomarkers. **b** The Kaplan-Meier (K-M) plot indicates that MIBC samples with a high level of CD8+ T cells have a better overall survival profile than those with low CD8+ T cells. **c** The K-M plot indicates that MIBC samples with a high level of TMB have a better overall survival profile than those with low TMB. **d** Distribution of CD8+ T cells among the molecular subtype, age, gender, stage, grade, and primary therapy response groups in the TCGA dataset. **e** Distribution of TMB among the molecular subtype, age, gender, stage, grade, and primary therapy response groups in the TCGA dataset. **f** The graphic depicting the relative change in area under the CDF curve, which allows determining the optimal K of 3 by first “elbow” rule. **g** The consensus matrices with samples as both rows and columns, ranging from 0 (never clustered together) to 1 (always clustered together)

Clinical characteristics of 403 patients with muscle-invasive bladder cancer (MIBC) used in this study were introduced in Table S1. The heatmap plot demonstrated that the expression patterns of immune checkpoint molecules (e.g. *PD-1*, *PD-L1*, *CTLA-4*, *HAVCR-2*, and *LAG-3*) distinguished the basal and luminal subtypes and that the basal tumors expressed higher levels of immune checkpoint molecules than luminal subtype (Fig. 1a). The Kaplan-Meier (K-M) survival analysis showed that TMB and CD8+ T cells were substantially associated with the MIBC prognosis (Fig. 1b,c). To be specific, the higher level of CD8+ T cells and TMB are associated with improved overall survival (OS). Simultaneously, we observed that B cells, dendritic cells, macrophages, neutrophils, NK cells, and CD4+ T cells were not statistically significantly correlated with the MIBC prognosis (*P* < 0.01) (Fig. S1A-F). Univariate and multivariate Cox regression analyses further confirmed that CD8+ T cells (HR = 0.664, 95% CI = 0.489–0.902, *P* = 0.008) and TMB (HR = 0.679, 95% CI = 0.500–0.921, *P* < 0.0129) were significantly associated with the OS, indicating again CD8+ T cells and TMB are independent prognostic predictors for the survival of MIBC patients. Besides, age (HR = 1.033, 95% CI = 1.017 to 1.049, *P* < 0.001), pathological stage (HR = 2.380, 95% CI = 1.596–3.548, *P* < 0.001), and molecular subtype (HR = 0.586, 95% CI = 0.426–0.805, *P* < 0.001) were found to be independent prognostic predictors (Table S2).

### Correlation analysis of CD8+ T cells and TMB

CD8+ T cells and TMB are nonidentical when comparing the median value of the clinicopathological characteristic groups of MIBC patients. Patents with high CD8+ T cells, as shown in Fig. 1d, were characterized by male, high pathological stage, and response to primary therapy, whereas high TMB was characterized by luminal subtype, high grade, and response to primary therapy (Fig. 1e). Importantly, PCC analysis revealed that TMB and CD8+ T cells exhibited a weak association (*r* = 0.11, *P* < 0.05) (Fig. S2A). Through PCC analysis, we also observed that the correlation between CD8+ T cells and immune checkpoints, such as *PD-L1, CTLA-4, PD-1, HAVCR-2, LAG-3*, is pronounced compared with TMB (Table S3). Together, these results indicate that CD8 + T cells and TMB may be independent indicators and that CD8 + T cells likely have better predictive ability than TMB when predicting the response to immune checkpoint inhibitors in MIBC.

### Construction of MIBC immunotypes and their clinical prognosis

The hierarchical consensus clustering was performed on the TCGA dataset comprising 403 tumor samples and three attributes (molecular subtype, TMB, and CD8+ T cells), with key parameters as follows: reps = 100, innerLinkage = complete, clusterAlg = hc, maxK = 10, and distance = pearson. The optimum cluster number K of 403 samples was determined with delta area plots and consensus cumulative distribution function (CDF) plots. According to the first “elbow” rule, the relative change in area under CDF curve suggests the optimal K of 3 (Fig. 1f). Similarly, consensus CDF plots showed that the CDF curve tended to be flat when K = 3, indicating the optimal K of 3 (Fig. S2B). In addition, the consensus clustering matrix suggested that the number of samples in the three groups was relatively balanced, which is a desirable result for further comparative analysis (Fig. 1g).

**Fig. 2:**
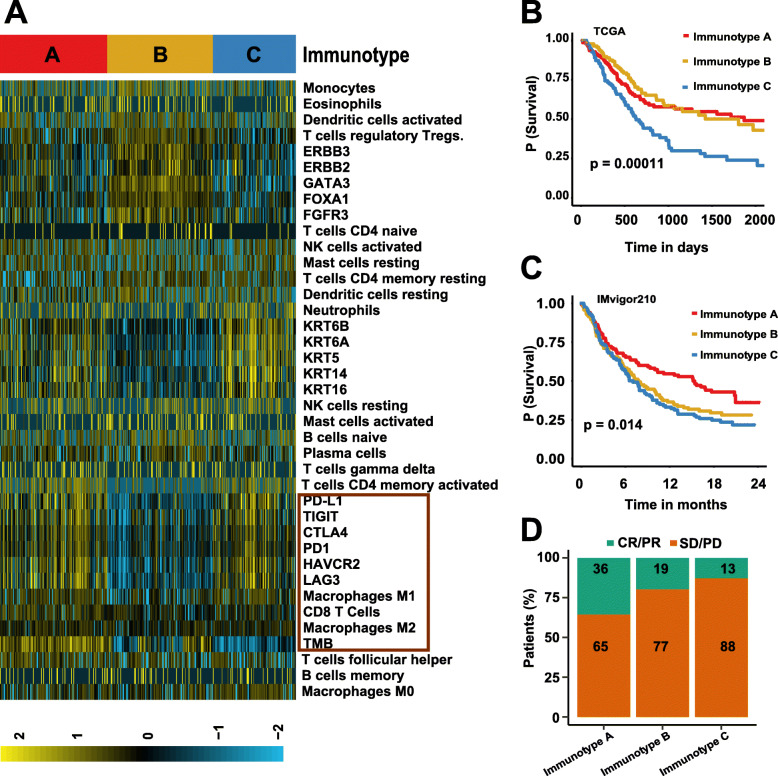
Molecular and clinical characterization of MIBC immunotypes. **a** The heatmap shows the distribution of basal, luminal, immune checkpoints, tumor-infiltrating immune cell-related biomarkers in immunotypes. **b** The K-M plot of patients based on TCGA dataset indicates that patients in immunotype A behaved a better overall survival profile than other immunotypes. **c** The Kaplan-Meier plot of patients based on the IMvigor210 dataset confirms that patients in immunotype A conferred the best overall survival profiles. **d** The bar graph depicts the response rate of MIBC patients to immunotherapy in the immunotypes based on the IMvigor210 dataset

A heatmap depicturing immune cells, immune check-point biomarkers, and TMB was provided, which clearly showed (i) immunotype A was characterized by the high expression of immunotherapy indicators such as immune checkpoint genes, CD8+ T cells, and TMB; (ii) immunotype B was characterized by low expression of immune checkpoint genes and CD8+ T cells and a moderate level of TMB; while (iii) immunotype C tends to express high immune checkpoint genes, moderate CD8+ T cells, and low TMB (Fig. 2a, Fig. S2C,D). The survival analysis revealed that the immunotype A conferred better overall survival (OS) and immunotype B conferred medium OS, while immunotype C exhibited a surprising poorer OS (*P* < 0.05, log-rank test) (Fig. 2b). Besides, immunotype A was significantly associated with better treatment outcomes, immunotype B was correlated with low histologic grade, while immunotype C was correlated with high tumor recurrence (Fig. S2E).

### Clinical outcomes of immunotypes in IMvigor210 cohort

The IMvigor210 cohort including 348 MIBC patients treated with Atezolizumab was used to further evaluate the clinical benefit of the immune subtypes. The baseline characteristics of the IMvigor210 cohort were described in Table S4. The K-M survival analysis has confirmed that immunotype A is associated with increased survival rate for patients treated with Atezolizumab, immunotype B is associated with medium survival rate, whereas immunotype C poorer OS (Fig. 2c). These findings are consistent with the results from the TCGA cohort, suggesting again that it is rather appropriate to use our method to distinguish patients.

It is to be noted that the objective response rate (ORR) was defined as the proportion of patients who have a partial or complete response to therapy. We found that patients in immunotype A behaved better ORR to Atezolizumab, about 36%. In addition, immunotype B exerted moderate ORR, about 19%, whereas immunotype C worst ORR, about 13% (Fig. 2d). Our results indicated that the efficacy of immunotherapy is strongly influenced by the molecular subtypes, immune checkpoints, and the composition and abundance of CD8+ T cells and TMB in the tumor microenvironment. In addition, we built a user-friendly model, named as ‘rfPI’ (Random Forest to Predict Immunotypes), for predicting the immunotype of MIBC patients, and it can be freely accessed from https://immunotypes.shinyapps.io/shiny/. The input data is a data frame, which contains the expression level of 736 immune-related genes. The output for rfPI will be: i) the predicted immunotype; ii) the predicted response rate for immunotherapy; iii) the mutated genes.

### Identification of mutated genes in immunotypes

**Fig. 3:**
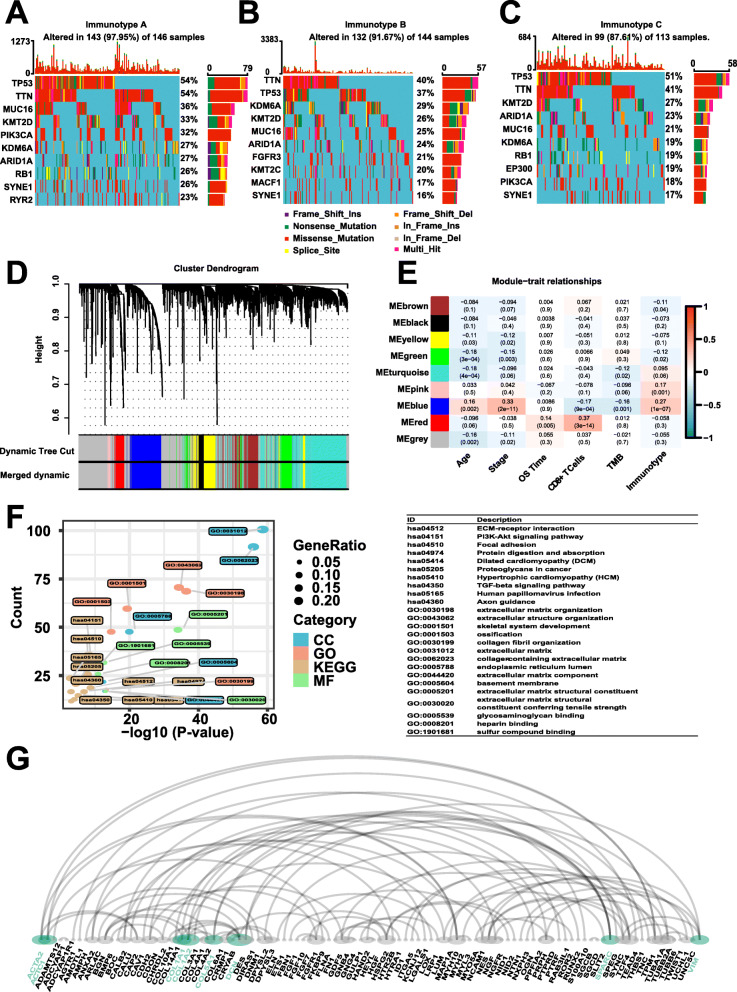
Genetic, transcriptome, and epigenetic characterization of MIBC immunotypes. **a-c** Genetic alterations in immunotype **a, b**, and **c**. **d** Dendrogram generated using the WGCNA shows nine highly parsimonious modules **e** PCC matrix between MEs and clinical traits. Each row corresponds to a module eigengene, each column corresponds to a trait, and each cell consists of the corresponding correlation and *P*-value. Among them, the blue module was the most relevant to the MIBC immunotypes. **f** Bubble plots for both enriched 10 KEGG pathways and 15 GO terms, for the blue module. **g** The protein-protein interaction network for genes within the blue module, and the green nodes represent the eight hub genes we identified

Genetic mutation is the basis of phenotype diversity among immunotypes. We sought to explore the potential regulatory tumor mutation genes among immunotypes. The mutation annotation file (MAF) of the patients with MIBC in TCGA dataset was analyzed using the R package “maftools”, and the summary of overall mutation profile was illustrated (Fig. 3a-c). *TTN*, *TP53*, *KMT2D*, *MUC16, ARID1A, KDM6A*, and *SYNE1* had a high frequency mutation rate in all immunotypes (> 16%). There are immunotype-specific genes differentially mutated as follows: (i) *PIK3CA* and *RB1* were found to be mutated in immunotype A and immunotype C; (ii) *FGFR3, KMT2C*, and *MACF1* immunotype B; (iii) *RYR2* was observed to be mutated in immunotype A; (iv) and *EP300* immunotype C.

### Weighted Correlation Network Analysis (WGCNA)

Functional modules can, furthermore, reveal more effectively the consistent differences during MIBC tumorigenesis and progression. WGCNA was performed on 4677 prognostic-specific mRNAs. According to scale independence and mean connectivity plot, we picked “8” as the proper soft-thresholding power, which can raise co-expression similarity to achieve consistent scale-free topology (Fig. S2F,G). A cluster dendrogram revealing nine modules highly co-expressed was provided, in which each co-expression module was assigned by an arbitrary brilliant color for reference, and the non-co-expression group was designated as a gray color (Fig. 3d). The MEs based on the first principal component were calculated for each module to assess the association between modules and clinical information including immunotype. The results, as visualized in Fig. 3e, showed that the blue module containing 546 genes possessed the highest correlation with immunotype (*r* = 0.27, *P* < 0.01) as well as pathological stage (*r* = 0.33, *P* < 0.01).

### Analysis of enriched GO terms and KEGG pathways

We then investigated enriched pathways related to genes within the blue module by performing GO and KEGG enrichment analysis, and the top-ranked terms and pathways were visualized in Fig. 3f. Some Immune system-related terms and pathways were largely identified, including ECM-receptor interaction, PI3K-Akt signaling pathway, focal adhesion, TGF-beta signaling pathway, human papillomavirus infection, extracellular matrix organization, skeletal system development, collagen-containing extracellular matrix, endoplasmic reticulum lumen, extracellular matrix structural constituent, and glycosaminoglycan binding.

### Protein-protein network analysis

**Fig. 4:**
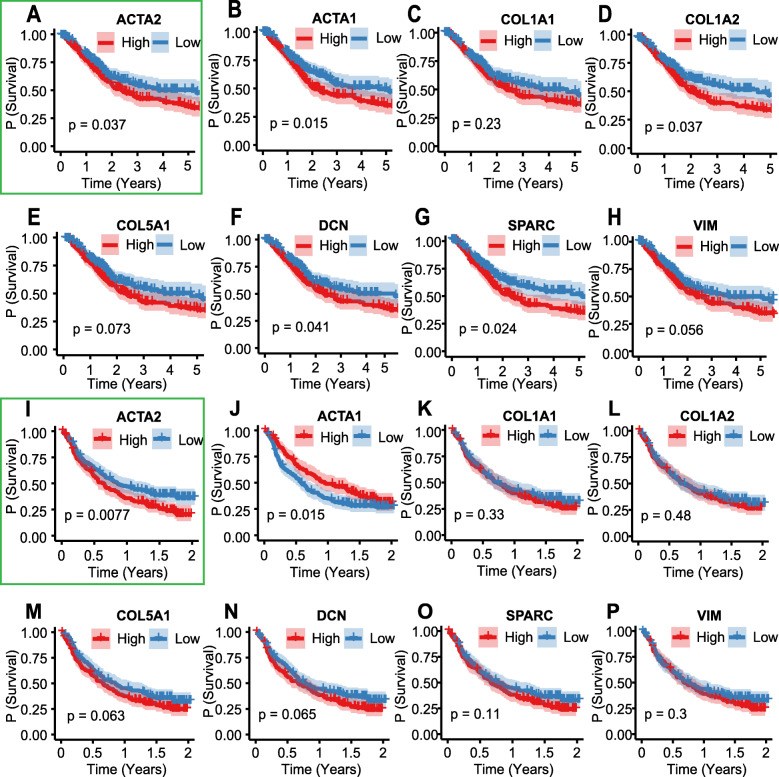
Kaplan–Meier survival analysis. K–M plot for eight hub genes, as to TCGA (**a**-**h**) and IMvigor210 dataset (**i**-**p**). The Kaplan-Meier plots confirmed that patients with low expression of the *ACTA2* genes, as shown in the green frame, behave a better overall survival profile

Cytoscape is an open-source and user-friendly software platform for visualizing molecular interaction networks and biological pathways. The experimental PPI between those 546 genes, retrieved from the STRING database, was visualized as a PPI network by Cytoscape software. Subsequently, eight hub genes from the network were identified based on the cut-off value of MCC score > 5 (Fig. 3e). The Kaplan-Meier (K-M) survival analysis for eight genes was conducted on both TCGA and the IMvigor210 datasets to determine which of them is of prognostic value indeed. Merely the low expression of *ACTA2*, as shown in Fig. 4, was associated with the survival benefit for MIBC patients from both TCGA and IMvigor210 datasets.

## Discussion

Although several clinical trials demonstrate that immune checkpoint inhibitors have been used with great success in the treatment of muscle-invasive bladder cancer (MIBC), the ORR in patients receiving Atezolizumab is quite low, about 14.8% for the entire study population [28]. It is one of the key challenges facing doctors today to stratify patients into subtypes that can obtain dramatic responses to drugs that target *PD-1*/*PD-L1* [7, 29, 30]. To tackle the above problem, MIBC immunotypes improving the ORR based on the multi-immunotherapy indicators were constructed using computational analysis.

CD8+ T cells play a major role in cancer immunity through their capacity to kill malignant cells upon recognition by T cell receptor of specific antigenic peptides presented on the surface of target cells by human HLA-I/β2M complexes [31]. Despite contributions by other immune cell subsets, CD8+ T cells have emerged as a predominant effector in most cancer immunotherapy settings, and many immunotherapeutic strategies are devoted to stimulating, enhancing, and maintaining responses by tumor-reactive CD8+ T cells [32]. TMB has been described as a powerful predictor of tumor behavior and response to immunotherapy, and it is independent of *PD-L1* expression in patients with small-cell lung cancer [33]. However, disease-specific TMB thresholds for effective prediction of response in various other malignancies are not well established [34]. Apart from some correlation analysis, the underlying mechanism behind TMB predicting sensitivity to immunotherapies is largely unknown [34]. Besides, molecular subtypes may provide independent and complementary information for predicting immunotherapy response. Several studies have implicated that basal and luminal subtypes are derived from distinct progenitor cells and the basal subtype has a higher ORR in the treatment of immunotherapy [7, 17, 18]. We demonstrated that the basal subtype exerted a totally different expression pattern in immune checkpoint genes as compared to the luminal subtype (Fig. 1a). Moreover, based on immune biomarkers such as CD8+ T cells, *PD-L1*, and TMB, the classification of human cancer into different immune types was recently proposed [7, 13, 35–37]. Herein, using the immunotherapy indicators comprising TMB, CD8+ T cells, and molecular subtype, we were able to stratify MIBC into three immunotypes, namely, immunotype A, immunotype B, and immunotype C.

The patients in immunotype A conferred the highest ORR and expressed a high level of immune checkpoints, TMB, and CD8+ T cells, designating that those patients were highly recommended to receive immunotherapy. It is to the fact that immunotype A here resembles ‘hot tumors’ previously defined [38]. The patients in immunotype B showed lower ORR, and they expressed a low level of immune checkpoints and CD8+ T cells and a moderate level of TMB. Whether this pattern is similar to “cold tumors” characterized by failed T cell priming (low tumor mutational burden, poor antigen presentation and intrinsic insensitivity to T cell killing), still requires further study [38–40]. The treatment strategies for ‘cold tumors’ are converting them into ‘hot tumors’ by enhancing T cell responses via cancer stem cells (CSCs) vaccine or adoptive T cell transfer [38, 41]. On the other hand, patients in immunotype C showed the lowest ORR. Interestingly, they expressed high immune checkpoints, moderate CD8+ T cells, but low TMB, and this group of patients may be not appropriated for immunotherapy.

Seven genes (*TTN, TP53, KMT2D, MUC16, ARID1A, KDM6A,* and *SYNE1*) were commonly mutated across three immunotypes, thus getting recognized as cancer predisposition genes. There are another seven genes equally important: *PIK3CA, RB1, FGFR3, KMT2C, MACF1, RYR2*, and *EP300*. These genes are differentially mutated among three immunotypes, cultivating a more detailed and precise understanding of mutation rates of MIBC immunotype. The modules of a co-expression network are more stable than individual genes because the overall function of a module can remain the same when individual gene expression can be replaced by other genes with similar redundant functions [42].

Network analysis identified eight hub genes (*ACTA2, ACTA1, COL1A1, COL1A2, COL5A1, DCN, SPARC, VIM*) for the module associated with MIBC immunotype. Consistent with our results, pathological stage-related hub gene role of *COL1A1, COL1A2, COL5A1* were reported earlier by another group [43]. The hub gene role of *ACTA1* was found in prostate cancer [44] and the prognostic role of *ACTA1* was found in head and neck squamous cell carcinoma (HNSCC) [45]. The experimental study suggests that decorin (DCN) does not affect directly anti-tumoural immune response, yet is required for efficient invasiveness in vitro [46]. Hub gene role of *DCN*, associated pathological stage, has been reported in our previous study [42]. Studies on *DCN* knockout mice have established that a lack of *DCN* is permissive for tumor development, and it is regarded as a tumor suppressor gene [47]. A previous study identified a 3-gene panel of epigenetic biomarkers comprising VIM, which can accurately detect bladder cancer in urine sediments and discriminate it from renal epithelial tumors and prostate cancer [48]. In another study, the correlation between the methylation levels of *EOMES, HOXA9, POU4F2, TWIST1, VIM,* and *ZNF154* in urine specimens and bladder cancer recurrence surveillance was discovered by real-time PCR [49]. A previous study found that loss of *SPARC* was associated with an inflammatory phenotype of tumor-associated macrophages and fibroblasts, with concomitant increased activation of urothelial and stromal NF-κB and AP1 in vivo and in vitro [50]. In human bladder tumor tissues, the frequency and intensity of *SPARC* were inversely correlated with disease-specific survival. *SPARC* can reduce carcinogen-induced inflammation and accumulation of reactive oxygen species as well as urothelial cell proliferation, and gene expression of *SPARC* significantly correlated with matrix metalloproteinase-2 gene expression [50, 51]. The multi-faceted contextual roles of *SPARC* were discussed in detail in a previous review [52], and the overall hypothesis was that *SPARC* exhibited differential expression and functions in the bladder cancer microenvironment. *ACTA2* was previously found to be associated with the prognosis of bladder cancer [53]. Consistent with the earlier reports, in our study, the prognostic value of *ACTA2* was validated both in the TCGA dataset and the IMvigor210 dataset.

Of note, the immunotypes in the study are constructed based on the TCGA cohort which does not receive immune checkpoint therapy. Although IMvigor210, an independent cohort, treated with immunotherapy has been used, more clinical trials of immune checkpoint blockade in prospectively collected cohorts are necessary. Besides, instead of evaluating all kinds of lymphocyte, only the CD8+ T cell fraction was selected to construct immunotypes, which might potentially ignore the effect of other immunotherapy biomarkers such as NK cells and macrophage [54, 55]. The deeper investigation of the role of immunotype-specific KEGG pathways or gene ontology terms we identified, in the human immune system diagram, will be beneficial in the context of understanding the underlying immune mechanisms that respond to bladder cancer, and it will be taken into account in our further research.

In conclusion, our study takes full advantage of verified immunotherapy indicators such as molecular subtype, CD8+ T cells, and TMB to jointly stratify samples into distinct immunotherapy response subgroups, namely immunotype A, B, and C. Among them immunotype A releases best clinical outcome with ORR of 36%. Together these findings will provide a new avenue for emerging immunotherapy strategies.

## Supplementary Information

**Additional file 1: Figure S1.** Kaplan-Meier survival curves for overall survival with respect to six tumor-infiltrating immune cells (A-F).

**Additional file 2: Figure S2.** (A) Pearson’s correlation coefficient plus corresponding *p*-value, for CD8+ T cells and TMB. (B) Graphic shows the cumulative distribution functions of the consensus matrix for each K. The proper K value was selected as 3, according to when the CDF tends to be flat. (C-D) Comparison of mean of CD8+ T cells and TMB among three immunotypes, respectively. (E) Correlation of immunotypes with tumorigenesis-related clinical information in the TCGA cohort. (F-G) Scale independence and mean connectivity suggest the optimal soft-threshold power of 8.

**Additional file 3: Table S1.** Baseline characteristics of patients in the TCGA cohort. **Table S2.** Univariate and multivariate Cox regression analyses for OS in TCGA. **Table S3.** Correlation between TMB / CD8+ T cells and immune checkpoints. **Table S4.** Baseline characteristics of patients in the IMvigor210 cohort.

## Data Availability

The authors declare that the data and code that support the findings of this study are available upon request from gjliu0325@gmail.com.

## Abbreviations

MIBC: Muscle-invasive bladder cancer
TCGA: The Cancer Genome Atlas
TMB: Tumor mutational burden
CDF: Cumulative distribution function
RF: Random forest
WGCNA: Weighted gene co-expression network analysis
PPI: Protein-protein interactions
K-M: Kaplan-Meier
MCC: Matthews correlation coefficient
ORR: Objective response rate
KEGG: Kyoto Encyclopedia of genes and Genomes
GO: Gene Ontology
PCC: Pearson’s correlation coefficient
OS: Overall survival
MAF: Mutation annotation file
BLCA: Urothelial bladder cancer
MEs: Module eigengenes
