# Supplementary figures and images for: Defining muscle-invasive bladder cancer immunotypes by introducing tumor mutation burden, CD8+ T cells, and molecular subtypes
Source: Hereditas. 2021 Jan 2;158:1. doi: 10.1186/s41065-020-00165-7 (PMC7778803; doi:10.1186/s41065-020-00165-7)

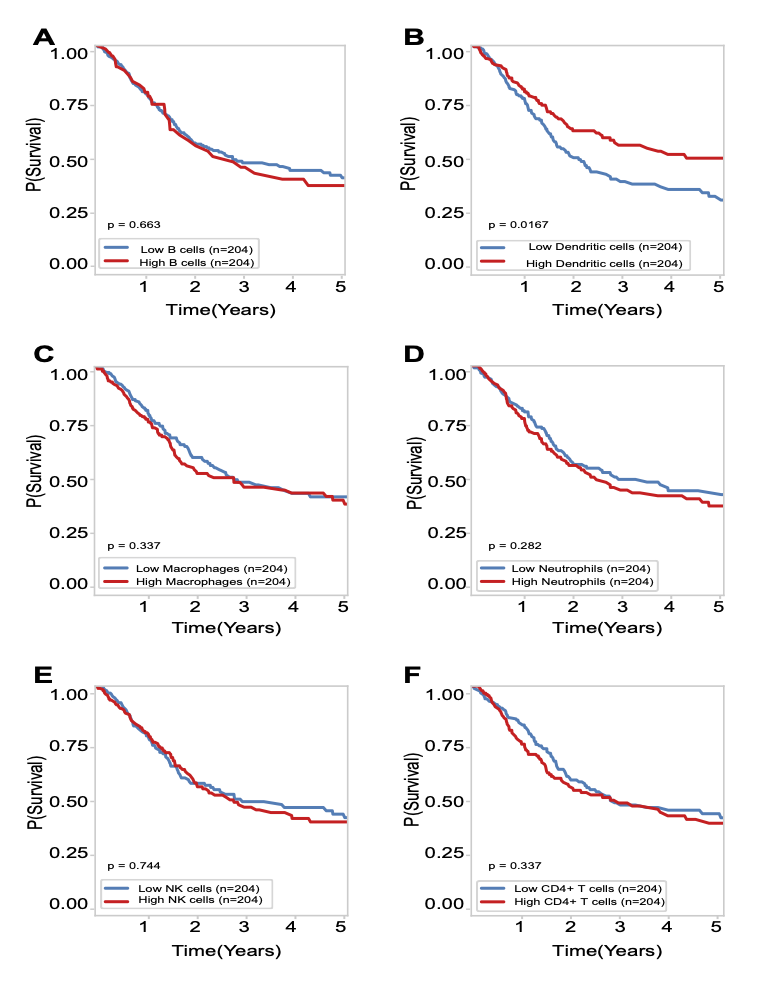

Supplement: Supplementary file 1 — Additional file 1: Figure S1. Kaplan-Meier survival curves for overall survival with respect to six tumor-infiltrating immune cells (A-F). [file 41065_2020_165_MOESM1_ESM.tiff]

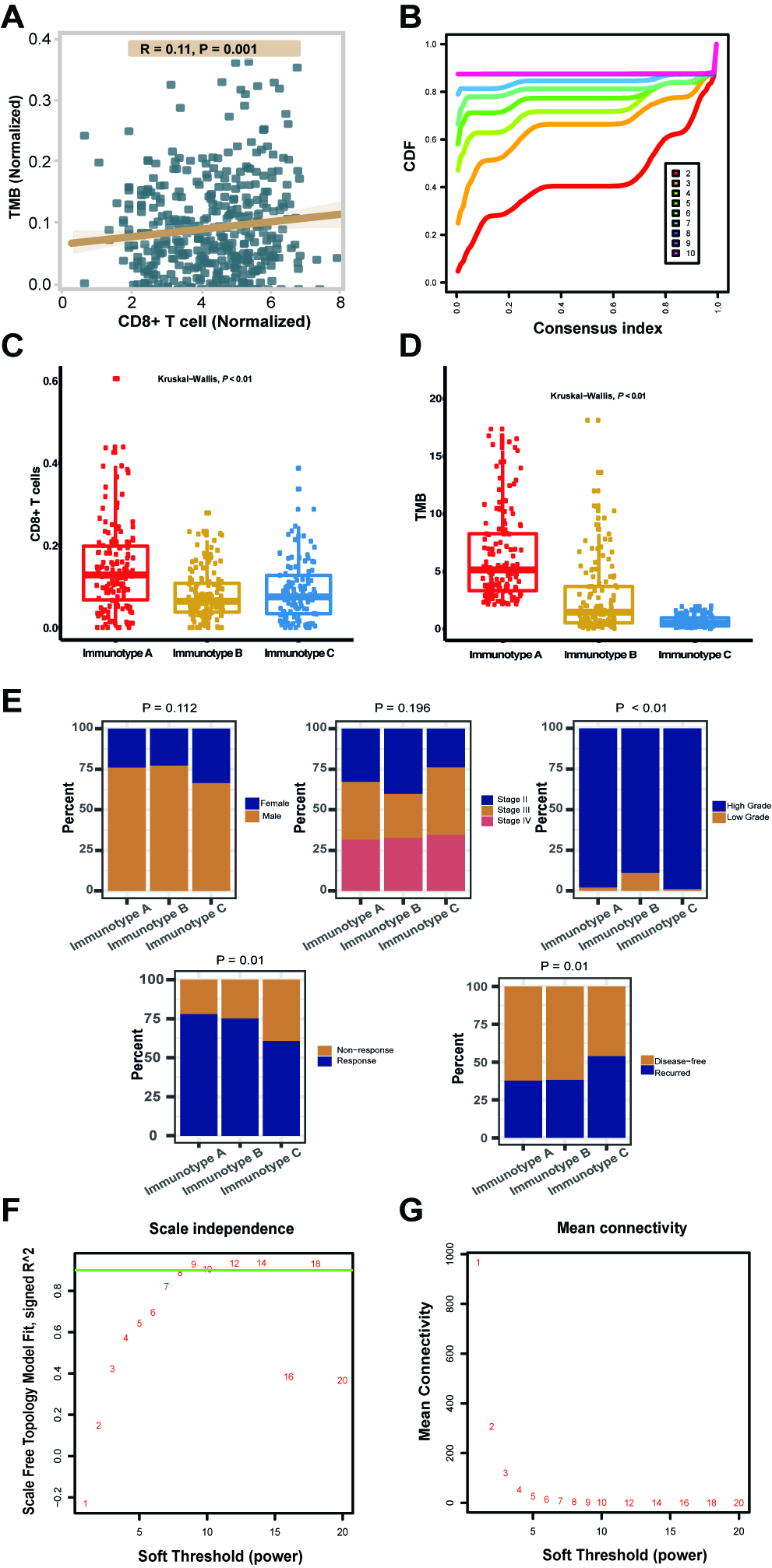

Supplement: Supplementary file 2 — Additional file 2: Figure S2. (A) Pearson’s correlation coefficient plus corresponding p-value, for CD8+ T cells and TMB. (B) Graphic shows the cumulative distribution functions of the consensus matrix for each K. The proper K value was selected as 3, according to when the CDF tends to be flat. (C-D) Comparison of mean of CD8+ T cells and TMB among three immunotypes, respectively. (E) Correlation of immunotypes with tumorigenesis-related clinical information in the TCGA cohort. (F-G) Scale independence and mean connectivity suggest the optimal soft-threshold power of 8. [file 41065_2020_165_MOESM2_ESM.tif]
