# Supplementary material for: Defining muscle-invasive bladder cancer immunotypes by introducing tumor mutation burden, CD8+ T cells, and molecular subtypes
Source: Hereditas. 2021 Jan 2;158:1. doi: 10.1186/s41065-020-00165-7 (PMC7778803; doi:10.1186/s41065-020-00165-7)
Supplement: Supplementary file 3 — Additional file 3: Table S1. Baseline characteristics of patients in the TCGA cohort. Table S2. Univariate and multivariate Cox regression analyses for OS in TCGA. Table S3. Correlation between TMB / CD8+ T cells and immune checkpoints. Table S4. Baseline characteristics of patients in the IMvigor210 cohort. [file 41065_2020_165_MOESM3_ESM.docx]

**Table S1.** Baseline characteristics of patients in the TCGA cohort.

| Traits | Clinical type | Sample (Rate) |
| --- | --- | --- |
| Histology type | Muscle Invasive | 403(100%) |
| Recurrence | Recurred  Disease-free | 170(42%)  233(58%) |
| Gender | Male  Female | 297(73%) 106(27%) |
| Stage | Stage II  Stage III  Stage IV | 133(33%)  138(34%) 132(33%) |
| Grade | High  Low | 383(95%)  20(5%) |
| Immunotype  Primary therapy outcome  Primary therapy outcome  Age | A  B  C  CR  PR  SD  PD  Not Available  Response  Non-response  Not Available  Older (>60)  Younger (<=60) | 146(36%)  144(36%)  113(28%)  230(58%)  22(5%)  29(7%)  68(17%)  54(13%)  252(63%)  97(24%)  54(13%)  296(73%)  107(27%) |

Note: CR = complete remission; PR = partial remission; SD = Stable Disease; PD = Progressive Disease.

**Table S2**. Univariate and multivariate Cox regression analyses for OS in TCGA.

| Traits | Univariate analysis  HR (95% CI) | *P*-Value | | Multivariate analysis  HR (95% CI) | | *P*-Value | |
| --- | --- | --- | --- | --- | --- | --- | --- |
| Age  Increasing  Gender  Male  Female  Stage  StageII  StageIII  StageIV  Subtype  Basal  Luminal  TMB  Lower  Higher  Grade  LowGrade  HighGrade  CD8+ T Cells  Lower  Higher | 1.031(1.016-1.047)  1 (reference)  1.118(0.808-1.546)  1 (reference)  1.585(1.039-2.417)  2.938(1.984-4.351)  1 (reference)  0.5391(0.395-0.734)  1 (reference)  0.6796(0.505-0.914)  1 (reference)  2.903(0.7183 -11.73)  1 (reference)  0.639(0.4745-0.8606) | | **<0.001**  0.501  0.0326  **<0.001**  **<0.001**  **0.0107**  0.135  **0.0031** | | 1.033(1.017-1.049)  1 (reference)  1.426(0.930-2.186)  2.380(1.596-3.548)  1 (reference)  0.586(0.426-0.805)  1 (reference)  0.679(0.500-0.921)  1 (reference)  0.664(0.489-0.902) | | **<0.001**  0.102  **<0.001**  **<0.001**  **0.0129**  **0.008** |

**Table S3.** Correlation between TMB / CD8+ T cells and immune checkpoints.

| Markers | Immune checkpoints | *R* | *P*-Value |
| --- | --- | --- | --- |
| TMB | PD-L1 | 0.0832 | 0.09091 |
|  | CTLA4 | 0.1353 | 0.005816 |
|  | PD-1 | 0.1544 | 0.001623 |
|  | HAVCR-2 | 0.1186 | 0.0158 |
|  | LAG-3 | 0.153 | 0.001798 |
|  |  |  |  |
| CD8+ T cells | PD-L1 | **0.3299** | **<0.001** |
|  | CTLA-4 | **0.3358** | **<0.001** |
|  | PD-1 | **0.4251** | **<0.001** |
|  | HAVCR-2 | **0.3203** | **<0.001** |
|  | LAG-3 | **0.4406** | **<0.001** |

Note: *R* = Pearson correlation coefficient (Strong correlation: 0.5-1; Medium correlation: 0.3-0.49; Low correlation: 0-0.29).

**Table S4.** Baseline characteristics of patients in the IMvigor210 cohort.

| Traits | Clinical type | Sample (Rate) |
| --- | --- | --- |
| Immunotherapy outcome | CR/PR  SD/PD  NA | 68(19%)  230(66%)  50(15%) |
| Gender | Male  Female | 272(78%)  76(22%) |
| Tobacco history | Previous  Never  Current | 197(57%) 116(33%)  35(10%) |
| Received Platinum | Yes No | 272(78%) 76(22%) |
| Immunotype | A  B  C | 116(33%)  116(33%) 116(33%) |

Note: CR = complete remission; PR = partial remission; SD = Stable Disease; PD = Progressive Disease; NA = Data is not available.
